# Phthalimide Derivatives as Anti-Inflammatory Agents: In Silico COX-2 Targeting and In Vitro Inhibition of PGE_2_ Production

**DOI:** 10.3390/pharmaceutics18010129

**Published:** 2026-01-20

**Authors:** Héctor M. Heras Martínez, Blanca Sánchez-Ramírez, Linda-Lucila Landeros-Martínez, David Rodríguez-Guerrero, José C. Espinoza-Hicks, Gerardo Zaragoza-Galán, Alejandro Bugarin, David Chávez-Flores

**Affiliations:** 1 Facultad de Ciencias Químicas, Universidad Autónoma de Chihuahua, Chihuahua 31125, Mexico; p288343@uach.mx (H.M.H.M.); bsanche@uach.mx (B.S.-R.); lilanderos@uach.mx (L.-L.L.-M.); a345673@uach.mx (D.R.-G.); jhicks@uach.mx (J.C.E.-H.); gzaragoza@uach.mx (G.Z.-G.); 2 Department of Chemistry and Physics, Florida Gulf Coast University, Fort Myers, FL 33965, USA

**Keywords:** phthalimide, in silico, molecular docking, PGE_2_, inhibition, production, anti-inflammatory

## Abstract

**Background/Objectives**: The development of specific inhibitors for cyclooxygenase-2 (COX-2) is a challenge for public health. A series of 17 *N*-phthalimide hybrids was evaluated using a functional M06 meta-GGA hybrid in combination with a polarized 6-311G (d, p) basis set. The top three candidates (**6**, **10**, and **17**) were synthesized and evaluated as selective COX-2 inhibitors of PGE-2 using an integrated in silico–in vitro approach. **Methods**: Molecular docking against COX-2 (PDB 5KIR) and COX-1 (PDB 6Y3C), supported by homology modeling and DFT geometry optimization (B3LYP/6-31G*), revealed that the phthalimide carbonyl groups and the 3,4,5-trimethoxyphenyl or geranyl-derived moieties establish key hydrogen bonds and hydrophobic contacts with Arg120, Tyr355, Tyr385, and Ser530 in the COX-2 active site, conferring predicted selectivity ΔGCOX−2 vs. COX−1 = −1.4 to −2.8 kcal/mol. **Results**: The compounds complied with Lipinski’s and Veber’s rules and displayed favorable ADMET profiles. In vitro assessment in LPS-stimulated J774A.1 murine macrophages confirmed potent inhibition of PGE_2_ production, 3.05 µg/mL, with compound **17** exhibiting the highest efficacy, 97.79 ± 5.02% inhibition at 50 µg/mL, and **10** showing 95.22 ± 6.03% inhibition at 50 µg/mL. Notably, all derivatives maintained >90% cell viability up to 250 µg/mL by resazurin assay and showed no evidence of cytotoxicity or mitosis potential in the tests at 24 h. **Conclusions**: These results demonstrate that strategic hybridization of phthalimide with natural and synthetic product-derived fragments yields highly potential PGE2 inhibitors. Therefore, compounds **6**, **10**, and **17** are promising lead candidates for the development of safer anti-inflammatory agents.

## 1. Introduction

Public health faces major global challenges, from immediate concerns to long-term issues, including the widespread use of pharmaceutical drugs, which affects millions of people worldwide each year [1,2]. Among these challenges, the search for novel nonsteroidal anti-inflammatory drugs (NSAIDs) with enhanced selectivity for specific inflammatory mediators or a pronounced cyclooxygenase 1 (COX-1) sparing effect remains a critical objective in modern medicinal chemistry [3]. Although NSAIDs have historically played an essential role in pain and inflammation relief, their clinical use is often constrained by gastrointestinal complications and cardiovascular risks, particularly with prolonged misuse or high-dose administration [4,5]. The development of NSAIDs with improved selectivity for pro-inflammatory pathways not only represents a paradigm shift in analgesic therapy but also holds the potential to significantly reduce adverse effects and alleviate public health burdens [6,7].

**Figure 1:**
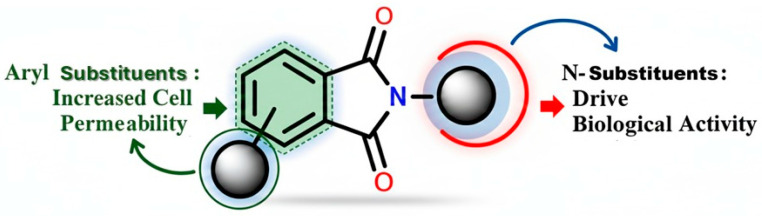
Phthalimide substituents and their physicochemical properties modification.

The continuous evolution of medicinal chemistry has driven the exploration of new biologically active molecules, among which phthalimide derivatives have demonstrated considerable anti-inflammatory potential [8,9]. These compounds have been employed in the design of prodrugs, inactive molecules that undergo biotransformation into therapeutically active NSAIDs within the body. This strategy enhances drug delivery while reducing off-target effects. Notably, the activation of phthalimide-based prodrugs may occur preferentially in inflamed tissues, where COX-2 is overexpressed, thereby improving therapeutic efficacy while minimizing toxicity. Additionally, the incorporation of organometallic compounds, *N*-heterocyclic carbenes (NHCs) [10,11], triazoles, and methoxy groups has emerged as a promising approach in the design of selective COX-2 inhibitors [12,13]. These chemical modifications have been shown to improve drug stability, bioavailability, and specificity, offering a refined approach to inflammation management, as shown in Figure 1.

Beyond their role in NSAID development, phthalimide derivatives are recognized pharmacophores due to their structural stability and biological versatility. They exhibit a broad spectrum of bioactivity, including anti-inflammatory [14,15,16], anticancer [17,18,19], anticonvulsant [20], and antimicrobial properties [21,22,23]. Historically, phthalimide-containing compounds have played a pivotal role in drug discovery, with commercially available drugs such as thalidomide [24], initially developed as a sedative, later repurposed for its potent anti-inflammatory and immunomodulatory effects, particularly in the treatment of multiple myeloma and erythema nodosum leprosum [18,19]. As shown in Figure 1, the phthalimide chemical structure exhibits electronic characteristics, such as the planar aromatic system and hydrogen-bond acceptor sites of the phthalimide core, which contribute to its strong interactions with biological targets, making it an attractive scaffold for the design of selective COX-2 inhibitors. Given the documented adverse effects associated with nonselective NSAIDs, particularly gastrointestinal toxicity, the development of COX-2-selective inhibitors based on phthalimide frameworks represents a promising strategy to enhance therapeutic outcomes while minimizing side effects. Recent studies highlight the potential of these derivatives in modulating inflammatory pathways, offering new perspectives for the development of safer and more effective anti-inflammatory agents [14]. The ongoing pursuit of novel NSAIDs is centered on optimizing the balance between COX-1 and COX-2 selectivity to tailor treatments to individual patient needs while reducing adverse effects. Additionally, research is increasingly focused on prodrugs and innovative drug delivery systems to improve therapeutic efficacy, further advancing the field of anti-inflammatory pharmacotherapy.

## 2. Materials and Methods

### 2.1. Homology Modeling

A combination of different computational methods was used to investigate the in silico binding modes and binding energies of the derivatives in cyclooxygenase (COX-1 and COX-2). The 3D structures of the macromolecules were built from homology modeling by using the crystal structure of *Homo sapiens*, oxygenation of an arachidonic acid site inhibitor to COX-2 (PDB ID: 5IKR) [25] and N-glycosylation by COX-1 (PDB ID: 6Y3C) [26]. These binding energies are important due to the narrow correlation with the principal source of the substrate pathway in prostaglandin production, which occurs in COX-2 and is expressed principally in the TYR385 and SER530 residues [27].

### 2.2. Computational Details

All molecules were studied using density functional theory (DFT) methods. quantum chemical calculations were performed at the B3LYP/6-31G* level of theory. DMSO was included as an implicit solvent using the PCM model to account for solvation effects, providing a realistic description of ligand behavior under experimental conditions. These calculations involved approximations in the hybrid meta-GGA functional M06. The functional M06 with its accuracy across the board for transition metals was determined, including the main group thermochemistry, the medium-range correlation energy, and the barrier heights, in combination with a 6-311G (d, p) polarized basis set. The computational optimization of phthalimide derivatives was performed employing the IEFPCM solvation method and DMSO as the solvent. All computational studies were performed using the software Gaussian 09 suite. The force constants and vibrational frequencies were determined by computing the analytical frequencies at the stationary points obtained after optimization, confirming the most stable conformations as true minima on the potential energy.

### 2.3. Docking Simulations

Preparation of receptor:

Protein structures were obtained from the Protein Data Bank (PDB) and prepared by adding missing hydrogen atoms and correcting protonation states according to standard protocols. The docking procedure was validated by redocking known ligands into the binding site, confirming that the method reproduces experimentally observed binding modes. Docking was performed in a semi-rigid manner, keeping the protein rigid while allowing flexibility for the ligands. Ligand structures were modeled in the gas phase. The cocrystallized structures of COX-1 and COX-2 [28] (PDB ID: 6Y3C and 5IKR, respectively) with a resolution of 2.4 Å were specifically chosen for the subsequent docking study, as previously detailed by Singh et al. in 2025 [29]. Prior to the docking analysis, structural refinements were made using PyMOL Molecular Graphic System (version 2.3.4) software. The cocrystallized heteroatoms, specifically atoms FLC to COX-1 [26] and COH, BOG, ID8, NAG and NH4 to COX-2 [25], were removed to maintain structural rigidity, as recommended. Furthermore, water molecules were eliminated using PyMOL. To enhance polar interactions and ensure molecular saturation, polar hydrogens were added using the Auto Dock Tool (ADT) [30]. This addition enhances bond stabilization. Subsequently, the refined structures were saved in PDBQT file format for subsequent analysis.

### 2.4. Drug-likeness Prediction

Molecular docking protocols are widely used to predict binding affinities for several ligands. In this work, we aimed to examine the possibility of an existing relationship between the experimental bioactivities of the inhibitors under study and the docking scores. To obtain accurate results, all docking experiments were performed with the default parameters: MSI Thin GF63 11UC-296MX laptop gaming with an Intel Core i5 Gen 11th processor (4.5 GHz), Graphics NVIDIA GeForce RTX 3050 4GB GDDR6, and 16 GB of RAM, running Windows 11.

Docking of the *N-*Phthalimide derivatives was performed using Auto Dock software (version 4.2) [30]. A model of the prostaglandin pathway channel open pore was used as a receptor. This open pore model was developed based on the homology model of the crystal structures. For our docking simulations, a cubic box with size 0.375 Å, number of points X-dimension 60, Y-dimension 46, and Z-dimension 44 centered at the center of mass X: −31.609 Y: −30.846 Z: 4.027 to COX-1 and X-dimension 58, Y-dimension 60, and Z-dimension 44, centered at the center of mass X: 43.617 Y: 0.906 Z: 63.757 to COX-2.

Schematic interaction diagrams were prepared using a multistep workflow. First, the docking complexes were generated and exported from AutoDock 4.2 in pdbqt format. These files were then converted into standard PDB format using Open Babel v3.1.1 [31], ensuring compatibility for downstream visualization. Subsequently, the PDB structures were processed in LigPlot v2.3 [32], which automatically generates two-dimensional representations of protein–ligand interactions. In these diagrams, hydrogen bonds are depicted as dashed lines, hydrophobic contacts are represented by radial arcs, and the amino acid residues involved are explicitly labeled. This approach enables a rapid and intuitive inspection of the binding environment, facilitating the comparison of multiple ligands within the COX-1 and COX-2 active sites. The resulting interaction maps provide critical insight into the structural determinants of ligand binding, supporting rational drug design and the optimization of phthalimide-based COX-2 inhibitors.

### 2.5. Chemistry

The *N*-phthalimide derivatives evaluated in this work (6, 10, and 17) were previously synthesized and fully characterized by our research group as potential TGF-β pathway inhibitors for anticancer therapy [17]. Their synthesis, purification procedures, structural confirmation by ^1^H-NMR, ^13^C-NMR, FT-IR, HRMS, elemental analysis, and purity assessment (≥98% by HPLC) are described in detail in the original report. All compounds were stored at 4 °C protected from light until use. For the present study, stock solutions (1 mg/mL) in supplemented DMEM in 0.1% DMSO were freshly prepared at the corresponding concentrations.

### 2.6. Pharmacology

Cell culture

The bioassays were performed according to the published procedure by Rodríguez-Castillo et al. [33]. The murine macrophage line J774A.1 (MOs; TIB-67 ATCC^®^, Rockville, MD, USA) was cultured with DMEM (Sigma-Aldrich^®^, St. Louis, MO, USA) supplemented with 10% *v*/*v* fetal bovine serum (FBS Gibco^TM^; Thermo Fisher Scientific, Inc., Waltham, MA, USA), 1% penicillin–streptomycin (10 mg/mL, Sigma-Aldrich^®^) and gentamycin (10 μg/mL, Sigma-Aldrich^®^, St. Louis, MO, USA). The cells were incubated at 37 °C with 5% CO_2_ (95% humidity) and harvested by scraping.

Evaluation of the Cytotoxicity of Phthalimide Derivatives to LPS-Induced MOs

The cytotoxic effects of the synthesized phthalimide derivatives on cell viability were evaluated using the resazurin reduction colorimetric assay in 96-well microplates, following established protocols [33]. Briefly, 1 × 10^5^ cells were seeded in high-glucose DMEM and exposed to compounds 6, 10, and 17 from our previously reported and characterized series [17]. The tested concentrations were 5, 10, 20, 40, 80, 100, and 250 µg/mL dissolved in DMEM supplemented with 0.1% dimethyl sulfoxide (DMSO; Sigma–Aldrich^®^, St. Louis, MO, USA). In addition, the precursor compounds were tested, including chrysin 13 µg/mL [34], geraniol 69 µg/mL [35], 3,4,5-trimethoxybenzoyl chloride 30 µg/mL [36], and potassium phthalimide salt at 37 µg/mL [37], which were analyzed at concentrations previously reported. Untreated cells served as the negative control, while 0.1% DMSO was used as the vehicle control. Curcumin (15 µg/mL; Sigma-Aldrich^®^, St. Louis, MO, USA) was used as the positive cytotoxic control. The cultures were incubated for 24 h at 37 °C in a humidified atmosphere containing 5% CO_2_. After 20 h of incubation, 2 µL of resazurin solution (6.5 µM) was added to each well and then incubated for an additional period to allow complete dye reduction. Absorbance was then measured at 590 nm using a Varioskan^®^ Flash microplate reader (Thermo Scientific^®^, Waltham, MA, USA). Cell viability was calculated according to the following equation:

(1) $$\mathrm{Viability}\;(\%)=\left(\frac{\mathrm{Absorbance}\;\mathrm{of}\;\mathrm{treated}\;\mathrm{cells}}{\mathrm{Absorbance}\;\mathrm{of}\;\mathrm{control}\;\mathrm{cells}}\right)\times 100$$

Evaluation of the effects of phthalimide derivative treatments on PGE2 production in LPS-stimulated macrophages.

A total of 3 × 10^6^ J774A.1 macrophages were seeded in 96-well microplates using supplemented DMEM (Gibco^TM^; Thermo Fisher Scientific, Waltham, MA, USA) and stimulated with 5 µg/mL lipopolysaccharide (LPS, type 0111:B4 from *Escherichia coli*; Sigma–Aldrich^®^, St. Louis, MO, USA). The treatments included test compounds 6, 10, and 17, as well as precursor compounds phthalimide, chrysin, 3,4,5-trimethoxybenzoyl chloride, and geraniol, at final concentrations of 50, 65, 50, 37, 13, 30, and 69 µg/mL, respectively. Cells incubated with 5 µg/mL LPS alone served as the inflammatory positive control, while cultures supplemented with 1.1 µM celecoxib (Pharmalife^TM^, Zapopan, Jal, Mex) or left untreated were used as the anti-inflammatory positive and negative controls, respectively. Following 24 h of incubation, the culture supernatants were collected and stored at −20 °C for further biochemical analysis.

PGE_2_ Isolation and Purification

Following treatments, culture supernatants were collected for the isolation of prostaglandin E_2_ (PGE_2_). Each sample was combined with 0.5 mL of a water:ethanol solution (1:4, *v*/*v*) and acidified by adding 10 µL of glacial acetic acid. The mixtures were gently vortexed, incubated at room temperature for 3 min, and subsequently centrifuged at 12,000 rpm for 2 min. The resulting supernatants were extracted using solid-phase using C18 cartridges (Sep-Pak C18, Waters, Milford, MA, USA) attached to a vacuum manifold system (Visiprep 24™ DL, Sigma–Aldrich, St. Louis, MO, USA). Prior to sample loading, the cartridges were conditioned with 3 mL of methanol followed by 3 mL of acidified water. Then, the columns were sequentially washed with 3 mL of acidified water and 3 mL of hexane. PGE_2_ was eluted with 1.5 mL of ethyl acetate, and the eluates were evaporated to dryness overnight at a controlled temperature of 17 °C in a Labnet 211d Stainless Steel incubator. The sample-dried residue was reconstituted in a volumetric flask to a final volume of 1 mL using a solution of acetonitrile:methanol:water 0.1% TFA (30:10:60, *v*/*v*/*v*), and the resulting mixture was sonicated at 17 °C for 15 min in a Branson M3800 ultrasonic bath operating at 110 W.

### 2.7. HPLC Analysis

UHPLC analysis was performed on a Thermo Scientific™ Ultimate 3000 system equipped with a DAD detector using an Agilent Poroshell 120 C18 column (2.7 µm, 150 × 4.6 mm). The injection volume was 50 µL, the column temperature was 30 °C, the autosampler was at 4 °C, and the mobile phase was acetonitrile:methanol:water 0.1% TFA (30:10:60, *v*/*v*/*v*). Chromatographic separation was monitored at 205 nm over a total run time of 20 min, and the calibration curve was carried out using the PGE_2_ (Sigma–Aldrich^®^, St. Louis, MO, USA) standard 50 µg/mL with R^2^ = 0.9999, which indicated that the model showed accuracy in calculating results within the dataset used to build it.

## 3. Results

**Figure 2:**
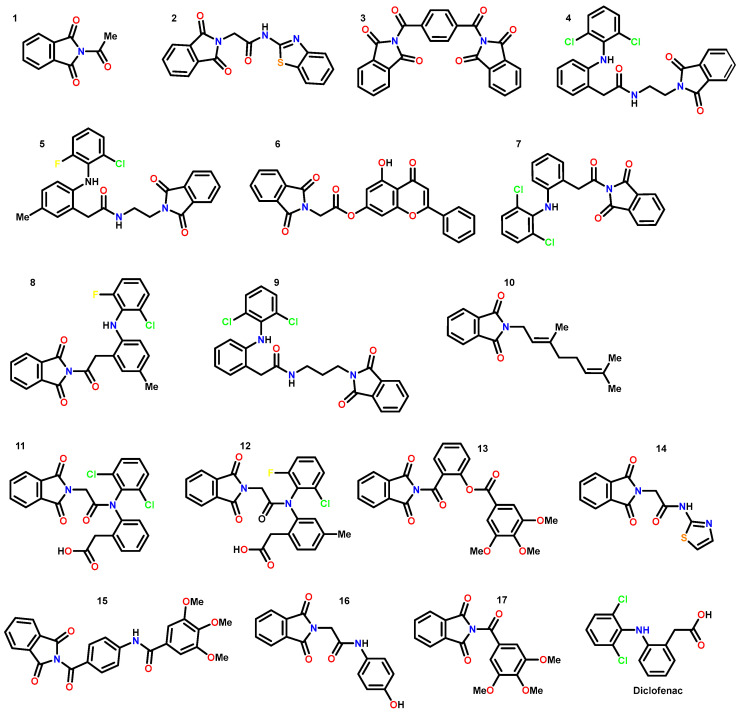
Chemical structures of phthalimide derivatives **1**: 2-acetylisoindoline-1,3-dione, **2**: *N*-(benzo[d]thiazol-2-yl)-2-(1,3-dioxoisoindolin-2-yl)acetamide, **3**: 2,2′-terephthaloylbis(isoindoline-1,3-dione), **4**: 2-(2-((2,6-dichlorophenyl)amino)phenyl)-*N*-(2-(1,3-dioxoisoindolin-2-yl)ethyl)acetamide, **5**: 2-(2-((2-chloro-6-fluorophenyl)amino)-5-methylphenyl)-*N*-(2-(1,3-dioxoisoindolin-2-yl)ethyl)acetamide, **6**: 5-hydroxy-4-oxo-2-phenyl-4H-chromen-7-yl 2-(1,3-dioxoisoindolin-2-yl)acetate, **7**: 2-(2-(2-((2,6-dichlorophenyl)amino)phenyl)acetyl)isoindoline-1,3-dione, **8**: 2-(2-(2-((2-chloro-6-fluorophenyl)amino)-5-methylphenyl)acetyl)isoindoline-1,3-dione, **9**: 2-(2-((2,6-dichlorophenyl)amino)phenyl)-*N*-(3-(1,3-dioxoisoindolin-2-yl)propyl)acetamide, **10**: (*E*)-2-(3,7-dimethylocta-2,6-dien-1-yl)isoindoline-1,3-dione, **11**: 2-(2-(*N*-(2,6-dichlorophenyl)-2-(1,3-dioxoisoindolin-2-yl)acetamido)phenyl)acetic acid, **12**: 2-(2-(*N*-(2-chloro-6-fluorophenyl)-2-(1,3-dioxoisoindolin-2-yl)acetamido)-4-methylphenyl)acetic acid, **13**: 2-(1,3-dioxoisoindoline-2-carbonyl)phenyl 3,4,5-trimethoxybenzoate, **14**: 2-(1,3-dioxoisoindolin-2-yl)-*N*-(thiazol-2-yl)acetamide, **15**: *N*-(4-(1,3-dioxoisoindoline-2-carbonyl)phenyl)-3,4,5-trimethoxybenzamide, **16**: 2-(1,3-dioxoisoindolin-2-yl)-*N*-(4-hydroxyphenyl)acetamide, **17**: 2-(3,4,5-trimethoxybenzoyl)isoindoline-1,3-dione and NSAID diclofenac.

Using AutoDock 4.2 [30] as modeling software, 17 phthalimide derivatives (Figure 2) were coupled to the active site, where the conversion of arachidonic acid to thromboxane and prostaglandins can occur in two isoforms: COX-1 and COX-2 [28,38]. Based on binding energy, the ligands were ranked. The results in Table 1 show the binding energies. Molecules **4** and **13** give satisfactory coupling energy values below −8.0 kcal/mol, which makes them good antagonists due to their hydrogen bonds [7]. Among them, the binding energies of **5**, **9**, **11**, and **12** were even better, with values under −8.5 kcal/mol, which makes them excellent candidates. These *N*-substituted phthalimide derivatives were classified into three groups based on the mean binding energy. Molecules **5**, **9**, **11**, **12**, and **15** belong to the group of major potential drug likeness, **3**, **4**, **6**, **8**, **10**, **13**, and **17** belong to the group of medium potential, and the remaining **1**, **2**, **7**, **14**, and **16** belong to the group of minor or null potential. Theoretically, all seventeen phthalimide analogs showed good binding energy compared to the reference drugs (celecoxib and diclofenac) previously reported by Nima et al. 2021 [39] and Abdel et al. [40]. All compounds showed good chemical interactions with the enzyme active site, mostly through hydrogen bonds, but compounds **1**–**7**, **10**, **11**, and **13**–**17** formed chemical interactions with the active site in at least one amino acid residue from the catalytic triad of 5KIR [41]. Furthermore, the molecular docking data indicate that the binding energies of all the tested compounds were no less than those of meloxicam. Compared to celecoxib, diclofenac and etoricoxib compounds **11** and **12** have similar COX-2 binding energies and selectivities for the active pocket amino acid residues of the receptor molecule.

These phthalimide derivatives were designed considering their physicochemical characteristics and structure–activity relationships, which primarily rely on functional groups analogous to those found in existing anti-inflammatory agents. Figure 2 shows the chemical structures investigated in this study. This selection criterion allowed us to make preliminary predictions about the interaction types that might be encountered in computational and biological studies. Notably, the prevailing feature among these structures is the presence of hydrogen bond acceptor groups, primarily within the phthalimide moiety. This is exemplified by the presence of two carbonyl groups. Furthermore, the methoxy groups linked to the aryl portion of the compounds serve as exemplary hydrogen bond acceptors, and they contribute to the overall lipophilic properties of the molecules.

Conversely, those molecules originating from the diclofenac core also exhibit notable lipophilic character, attributable to the presence of fluorine and chlorine halides. In contrast, the results presented in Table 1 and Table 2 demonstrated that compounds containing hydrogen bond donor groups, such as amides, aromatic amines, and hydroxyls, display limited or negligible affinity toward COX-2. This phenomenon is consistent with the specific features of the active sites in question. Furthermore, the carbonyl groups of the phthalimide scaffold furnish heightened binding energy through the formation of stable hydrogen bonds, exhibiting a pronounced affinity for residues within the catalytic triad, ARG120, TYR351 and SER530. Our analysis reveals comparable or even superior pharmacophore properties in comparison to the reference compounds celecoxib, diclofenac and etoricoxib.

**Figure 3:**
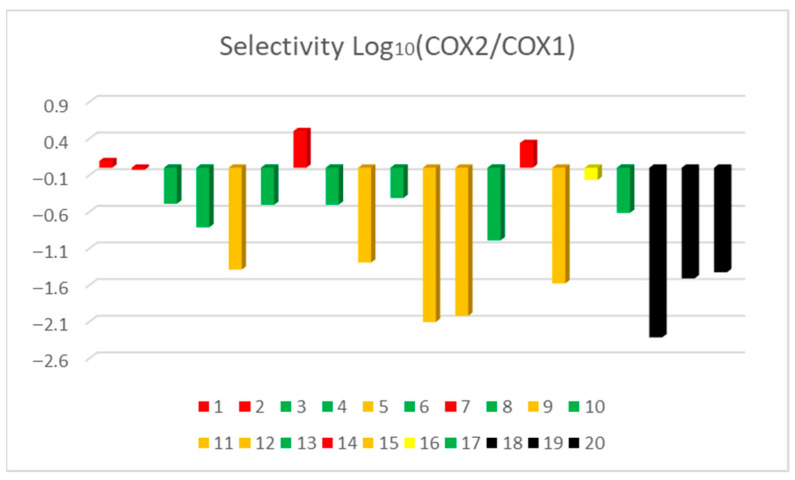
Selectivity COX-2 vs. COX-1 of compound (**1**–**17**) and reference controls; celecoxib (**18**), diclofenac (**19**), and etoricoxib (**20**). Bright yellow bars: compounds exhibiting the highest predicted selectivity for COX-2 (most negative Log_10_ values). Green bars: compounds with intermediate selectivity for COX-2. Pale yellow bars: compounds displaying low selectivity for COX-2. Red bars: compounds showing higher predicted affinity for COX-1 or negligible/no selectivity for COX-2. Black bars: (celecoxib, diclofenac, and etoricoxib) for direct benchmarking.

Table 1 illustrates the discernible influence of chemical structures and their associated functional groups in establishing a specific affinity toward either COX-1 or COX-2. Notably, larger molecular entities with increased molecular weights predominantly exhibit heightened affinity for COX-2. As expounded by Krzyżak et al., 2020 [41], the differential pore size between COX-2 and COX-1 stands as a pivotal and utilitarian determinant, facilitating the identification of promising candidates with augmented anti-inflammatory potential, alongside considerations of relative selectivity. As previously elucidated by González et al., 2002 [42], the COX-1 active site predominantly exhibits polar characteristics. Conversely, the COX-2 active site, in addition to its larger pore size, displays a pronounced lipophilic environment. These observations furnish valuable insights into the molecular determinants governing the selectivity and affinity of the compounds under study for COX-1 and COX-2, as shown in Figure 3 thereby enhancing the prospects of rational inhibitor design with potential therapeutic implications. This scientific rendition emphasizes the structural and functional attributes of the compounds, which impact their interaction with COX-1 and COX-2, guiding the rational design of enzyme inhibitors with therapeutic potential.

For instance, derivative **1**, characterized by its smaller molecular footprint, registers an affinity energy of 0.0911 toward COX-1, whereas the larger molecular structure of derivative **11** yields the highest documented affinity for COX-2, with an affinity energy of −2.1052. Analogous characteristics are evident among derivatives **5**, **9**, **12**, and **15**, sharing a remarkably consistent molecular weight within a ±5 kcal/mol range. These compounds encompass functional groups, including aryl chlorides, ester moieties, amides, and notably, carboxylic acids, which substantiate their pivotal role in fostering interactions with the respective cyclooxygenase enzymes. Among the evaluated derivatives, the most remarkable molecules exhibiting strong affinity for COX-2 (≤−0.8) were **4**, **5**, **9**, **11**, **12**, **13**, and **15**. Notably, their structural backbones are derived from established commercial anti-inflammatory agents, namely, diclofenac (**4**, **7**, **9**, and **11**) and lumiracoxib (**5**, **8**, and **12**), as well as **15**, which originates from the benzocaine-related synthon *p*-aminobenzoic acid [8]. This structural heritage appears to contribute significantly to their enhanced binding profiles.

In particular, derivatives **11**, **12**, and **15** outperformed diclofenac and lumiracoxib in terms of calculated COX-2 affinity, underscoring the impact of subtle modifications on their binding behavior. These compounds also demonstrated stronger and more specific interactions with the key catalytic triad residues ARG120, TYR355, and SER530, which are critically involved in prostaglandin PGE-2 biosynthesis. The presence of hydrogen-bond donor and acceptor groups in these derivatives facilitated the formation of stable hydrogen bonds within the COX-2 active site, thereby reinforcing the binding strength and selectivity. These findings suggest that structural refinement of known NSAID scaffolds, as exemplified by **11**, **12**, and **15**, holds considerable promise for the development of novel, selective COX-2 inhibitors with improved molecular recognition of the enzyme’s active site (Figure 3).

This complex interplay of molecular dimensions, functional groups, and concomitant enzymatic interactions is paramount in rational drug design, especially in the context of anti-inflammatory agents. In summary, this study is focused on highlighting the complex dynamics between chemical structure and biological activity in the realm of new anti-inflammatory agents. This approach to molecular design and the integration of functionally relevant moieties holds substantial promise for optimizing candidates with therapeutic potential for the management of inflammatory conditions. These considerations bear substantial significance in the realm of pharmaceutical research and medicinal chemistry, epitomizing the critical importance of a comprehensive understanding of compound chemistry in the quest for better NSAIDs.

Figure 3 depicts the COX-2 vs. COX-1 selectivity of the examined compounds. Derivatives **7** and **14** exhibited higher selectivity for COX-1, whereas derivatives **11**, **12**, and **15** showed superior selectivity for COX-2, even higher than both diclofenac and etoricoxib. Exploratory studies have examined the feasibility of combining phthalimide derivatives with other anti-inflammatory agents to achieve synergistic effects, potentially enhancing therapeutic efficacy while reducing required dosages and associated adverse effects. These findings further reinforce the relevance of phthalimide scaffolds in drug discovery, particularly in the development of more effective and safer anti-inflammatory therapies.

Based on the docking predictions, compounds **6**, **10**, and **17** were selected for biological evaluation in the J774A.1 (ATCC^®^ TIB-67™) macrophage line. The resazurin-based viability assays showed that none of the compounds induced cytotoxicity across the full concentration range tested. These results indicate that the phthalimide derivatives exhibit an adequate cellular safety profile. This is notable given that chemically similar scaffolds—particularly those with higher aromatic or lipophilic character—can negatively affect cell viability. Consistent with this, no morphological alterations or reductions in metabolic activity were observed in treated cells.

**Figure 4:**
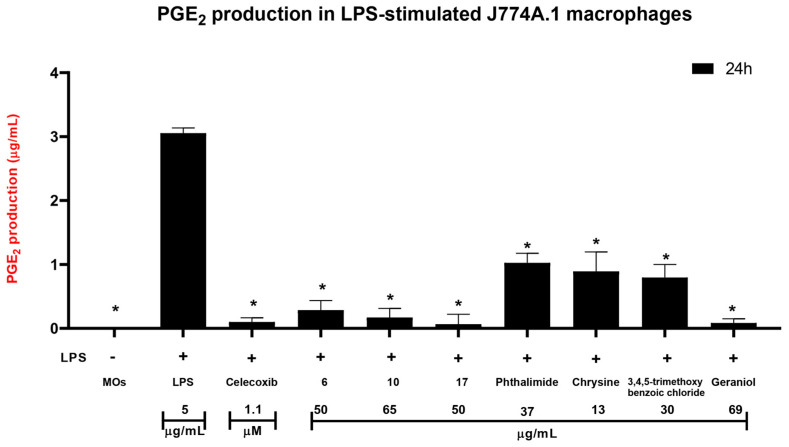
Effect of different treatments on PGE_2_ production in LPS-stimulated macrophages. Macrophages were stimulated with lipopolysaccharide (LPS, 5 μg/mL) to induce PGE2 production and treated with compounds (celecoxib, **6**, **10**, **17**, phthalimide, chrysin, trimethoxy, and geraniol). The MO group represents nonstimulated macrophages (basal control), while LPS served as positive control of inflammation. Bars represent the mean ± standard deviation (SD) of three independent replicates. A significant increase in PGE2 levels was observed in the LPS group compared to the basal control (MOs). In contrast, most treatments (celecoxib, **6**, **10**, **17**, phthalimide, trimethoxy, and geraniol) markedly reduced PGE2 production relative to LPS, indicating potential anti-inflammatory effects. Asterisks (*) denote statistically significant differences (*p* < 0.05) compared to LPS according to one-way ANOVA followed by Tukey’s post hoc test.

PGE_2_ quantification in LPS-stimulated macrophages (5 µg/mL) revealed a pronounced increase in prostaglandin levels compared with the basal control, confirming effective induction of an inflammatory state 8.65 µg/mL. As expected, treatment with celecoxib markedly decreased PGE_2_ production to 0.101 µg/mL, validating its role as a pharmacological positive control. Among the phthalimide derivatives evaluated, **6**, **10**, and **17** all produced a substantial reduction in PGE_2_ secretion after 24 h, yielding final concentrations of 0.284 µg/mL, 0.170 µg/mL, and 0.067 µg/mL, respectively. These values indicate that the derivatives significantly attenuated prostaglandin synthesis, with 10 and **17** displaying inhibitory magnitudes approaching those of celecoxib, shown in Figure 4.

The precursor molecules linked to phthalimide reduced PGE_2_ levels, but with varying 2degrees of effectiveness. The absolute concentrations (µg/mL) were: phthalimide 1.02, chrysin (5,7-dihydroxy-2-phenyl-4H-chromen-4-one) (chrysin) 0.893 µg/mL, ((*E*)-3,7-dimethylocta-2,6-dien-1-ol) geraniol 0.085 µg/mL, and 3,4,5-trimethoxybenzoyl chloride 0.797 µg/mL. Figure 4 shows both the absolute concentrations and the percentage reduction compared to the LPS control. Most treatments caused a statistically significant decrease in PGE_2_ (*p* < 0.05), confirming reproducible inhibition of LPS-induced prostaglandin synthesis. Compounds **10** and **17** had the strongest effects, which agrees with the inhibitory patterns predicted by the in silico analysis for the phthalimide scaffold. A percent-recovery test was performed using a 30 µg/mL PGE_2_ solution according to the established extraction protocol. This test was done to check the method’s accuracy, sensitivity, and reproducibility. The final measured concentration was 26.88 µg/mL, corresponding to a recovery of 89.61%, showing that the extraction succeeded.

To assess PGE_2_ stability in solution over time, a 5 µg/mL solution prepared in water (0.1% TFA):acetonitrile:methanol (60:30:10) was monitored at 1, 7, 14, 21, and 30 days. This experiment was done to see how stable PGE_2_ is under the experimental conditions. On day 30, only 4.48% degradation was observed, corresponding to a loss of 0.22 µg/mL, confirming that PGE_2_ stays stable throughout the workflow.

## 4. Discussion

A fundamental aspect of new drug development involves the identification and optimization of pharmaceutical candidates with high binding affinity for specific biological targets, such as key regulatory enzymes. The inhibition constant (*Ki*) serves as a quantitative parameter for evaluating and comparing the potency of enzyme inhibitors. In computational drug design, particularly through molecular docking, theoretical *Ki* values can be derived from the binding free energy (ΔG), providing a predictive measure of a compound’s interaction with the enzyme’s active site. Table 2 presents a comparative theoretical analysis of phthalimide derivatives against COX-1 and COX-2 enzymes, expanding upon the trends outlined in Table 1. The data aim to assess the relative binding affinities of each compound and their potential selectivity toward COX isoforms. In this context, a logarithmic linear model is employed to elucidate correlations between structural features and inhibitory potential. This modeling approach supports the rational design of enzyme-targeting agents, facilitating the selection of promising lead compounds and structural optimization to enhance specificity and activity, as shown in Figure 3.

**Figure 5:**
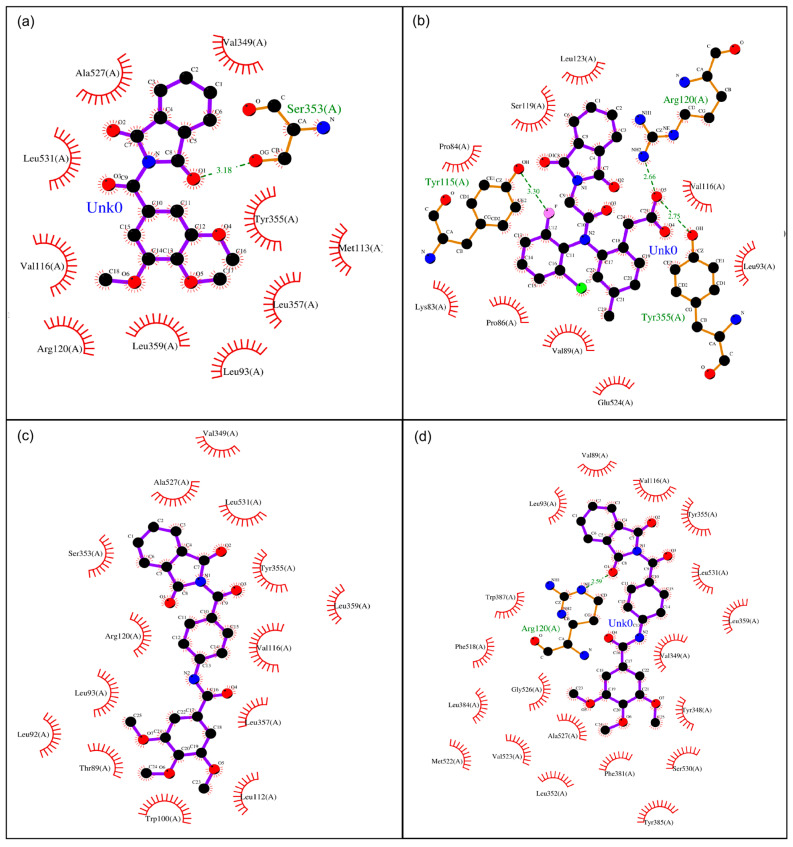
Docking ligand interactions with COX-1 and COX-2; (**a**) **12**—COX1, (**b**) **12**—COX2, (**c**) **15**—COX1, (**d**) **15**—COX2.

According to the Log_10_ linear model adopted by Park and Anthony Bavry 2014 [43], lower Ki values reflect a higher affinity for COX-2, while increasingly positive values indicate preferential binding to COX-1. As illustrated in Figure 3, compounds **1**, **2**, **7**, and **14** exhibit marked affinity toward the COX-1 active site. These molecules are characterized by molecular weights below 300 g/mol, with the exception of **7**, which remains under 450 g/mol. It is noteworthy that all derivatives from **1** to **17** comply with Lipinski’s rule of five, particularly in terms of acceptable molecular weight, as well as appropriate numbers of hydrogen bond acceptors and donors. These properties support their drug-likeness and further validate their potential as viable therapeutic candidates. On the other hand, compounds **12** and **15** displayed comparatively lower affinity for COX-2, as illustrated in Figure 5. Within the set of positive values, higher magnitudes correspond to stronger affinity toward COX-1. In contrast, compounds **1** and **2** yielded values close to zero, indicating negligible selectivity and no substantial difference in their interaction with COX-1 versus COX-2.

### Pharmacology

In silico analysis revealed derivatives with slightly higher selectivity for COX-2, mainly compounds **11** and **12**, based on Table 1. However, compounds **6**, **10**, and **17** were selected for experimental validation due to practical and feasibility criteria: (i) simple one- or two-step synthesis from low-cost and commercially available starting materials (phthalimide, geranyl bromide, 3,4,5-trimethoxybenzoyl chloride, and 5,7-dihydroxy-2-phenyl-4H-chromen-4-one (chrysin), with isolated yields >90% and purification limited to recrystallization without further purification by flash chromatography; (ii) favorable solubility profiles for the lumiracoxib-derived hybrid compounds **5**, **8**, and **12**, as the precursor could not be acquired; and (iii) complete prior spectroscopic and analytical characterization, ensuring reproducibility and direct identity confirmation [17]. These attributes allowed for the reliable execution of cell assays and dose–response studies, thus justifying their prioritization over candidates with greater theoretical selectivity but greater synthetic and handling complexity.

Based on the docking results, compounds **6**, **10** and **17** were tested in the J774A.1 (ATCC^®^ TIB-67™) macrophage line in LPS-stimulated conditions, and the results provided a clear indication of the anti-inflammatory potential of the phthalimide derivatives **6**, **10**, and **17**. As expected, exposure to LPS (5 µg/mL) markedly elevated PGE_2_ levels relative to the basal condition, confirming effective activation of the inflammatory pathway. Treatment with celecoxib significantly attenuated PGE_2_ synthesis, and a concentration of 1.1 µM was used by Sandhya Chahal et al. [7] in RAW 264.7 and J774A.1 cells, validating the experimental system and serving as an appropriate pharmacological benchmark. All three phthalimide derivatives produced a measurable reduction in PGE_2_ levels after 24 h of incubation, with **17** exhibiting the strongest inhibitory activity, followed by **10** and **6**. Although the magnitude of inhibition varied among the derivatives, the trend was consistent and reproducible across experiments. These findings suggest that the tested molecules are capable of interfering with prostaglandin biosynthesis pathways, likely through partial or indirect modulation of COX-2 activity.

The individual precursor compounds (phthalimide, 5,7-dihydroxy-2-phenyl-4H-chromen-4-one (chrysin), (*E*)-3,7-dimethyl-2,6-octadien-1-ol (geraniol), and 3,4,5-trimethoxybenzoic acid were evaluated alongside the hybrids to assess the relative contribution of each structural fragment to the observed anti-inflammatory profile. Chrysin alone showed only modest PGE_2_ inhibition in macrophage models, whereas derivative 6 achieved approximately 90% inhibition, indicating that linkage to the phthalimide scaffold markedly improves potency, likely through enhanced cellular uptake and/or metabolic stability. Similarly, geraniol exhibited strong activity, ~94% inhibition, which was maintained and slightly increased in derivative 10~95%, with the added benefit of lower cytotoxicity at high concentrations. Phthalimide and 3,4,5-trimethoxybenzoic acid displayed limited effects on their own, suggesting that the synergistic integration of lipophilic aromatic moieties with the phthalimide core drives the improved pharmacological profile. These comparisons highlight the value of the hybridization approach, demonstrating that the phthalimide scaffold not only preserves but frequently enhances the inherent bioactivity of the precursor fragments while improving overall biocompatibility.

Compared with similar structures, these findings are also consistent with previous reports describing the biological behavior of precursor molecules. Sassi et al. [44] demonstrated that chrysin exerts potent antitumor activity in vitro and in vivo using B16F10 melanoma cells, and the authors reported enhanced cytotoxic activity of macrophages, NK cells, and CTLs without evidence of toxicity to these immune populations, highlighting the compound’s favorable safety profile. Similarly, Park et al. evaluated a series of structurally modified chrysin derivatives in RAW 264.7 macrophages and found that halogenation, particularly bromine substitution and methylation of phenolic hydroxyl groups, improved anti-inflammatory performance, reducing PGE_2_ production by 99.9% and 99.2%, respectively. In contrast, unmodified chrysin showed only an 11.12% reduction according to Park H et al. [45]. In comparison, the present study showed a 70.74% decrease in PGE_2_ levels, indicating that the incorporation of the chrysin moiety into the phthalimide scaffold 6 enhances its bioactivity beyond the parent compound while maintaining excellent cellular compatibility. In contrast, (*E*)-3,7-dimethyl-2,6-octadien-1-ol (geraniol) has no reported anti-inflammatory assays in RAW 264.7 or J774A.1 cell lines, and only in cancer cell lines, 3,4,5-trimethoxybenzoic chloride, or similar structures, were reported to be cytotoxic.

The cell viability assays revealed that derivatives **6**, **10**, and **17** preserved >90% survival in J774A.1 macrophages across all tested concentrations, including the highest dose of 250 µg/mL, showing that the absence of detectable cytotoxicity prevented IC_50_ determination. This complete absence of cytotoxicity is particularly relevant because small structural modifications, especially those increasing aromaticity or lipophilicity, often raise the likelihood of nonspecific membrane disruption or mitochondrial imbalance. In contrast, all three hybrids maintained metabolic activity within the expected range for biocompatible molecules, indicating that phthalimide conjugation does not introduce deleterious effects under these conditions.

Importantly, no signs of mitotic dysregulation or morphological abnormalities were detected. The absence of proliferation defects aligns with previous findings showing that the precursor scaffolds do not induce abnormal immune cells. For instance, chrysin, the key aromatic fragment in derivative **6**, is known to selectively induce cell cycle arrest in tumor cells while preserving normal mitotic patterns in macrophages and hematopoietic stem cells through Nrf2 activation and ROS suppression [44,45,46]. Likewise, (*E*)-3,7-dimethyl-2,6-octadien-1-ol (geraniol) exhibited a remarkably strong inhibitory effect on PGE_2_ production, achieving a 94.49% reduction in LPS-stimulated macrophages. This pronounced activity indicates that the terpenoid precursor possesses an intrinsic ability to modulate inflammatory signaling, likely attributable to reported interactions with membrane-associated pathways and COX-2-related catalytic processes. Notably, compound **10**, which incorporates the geranyl moiety into the phthalimide scaffold through an amide linkage, produced a 95.22% inhibition, only marginally higher than that of geraniol alone. This close correspondence suggests that the dominant contribution to the biological effect arises from the geraniol fragment itself, while phthalimide conjugation may play a secondary or supportive role by improving physicochemical properties [35,47,48].

The trimethoxybenzoic acid motif in derivative **17** mirrors the behavior of related trimethoxy-substituted hybrids, which exhibit strong COX-2 inhibitory activity yet retain excellent biocompatibility in macrophage models of gastric cancer cells [36]. Alanazi et al. reported that structurally comparable trimethoxybenzoic-phthalimide hybrids showed potent anti-inflammatory activity with no evidence of cytotoxicity or mitotic abnormalities with IC_50_ = 0.18 µM surpassing celecoxib, together with an in vivo anti-inflammatory response, and comparable to compound **17** with 0.19 µM, an observation consistent with the results obtained in the present study [37]. These parallels strongly suggest that the trimethoxy aromatic system, when coupled to phthalimide, promotes molecular stability and target selectivity without compromising cell integrity.

The phthalimide scaffold itself may contribute to this profile. Numerous phthalimide-based molecules retain >90% viability in macrophage assays and do not induce chromosomal instability or mitotic errors [14,49]. Its incorporation appears to buffer the cytotoxic tendencies that individual precursors may exhibit in tumor cells while maintaining the capacity for anti-inflammatory activity. This structural behavior is consistent with reports showing that phthalimide conjugation often reduces nonspecific toxicity and improves pharmacological compatibility in immune cell models.

Overall, the combined evidence indicates that derivatives 6, 10, and 17 show no cytotoxicity and no adverse effects on cell viability or proliferation at 24 h. The high survival percentages and the absence of proliferation abnormalities reinforce the suitability of these hybrids for further biological evaluation and position them as promising molecules for anti-inflammatory drug development.

The cell viability values shown in Table 3 obtained across the treatments were close to or slightly above 100%, a behavior common in resazurin-based metabolic assays. Notably, compounds **6** and **10** produced a concentration-dependent increase in metabolic activity, reaching up to ~21% above the control at 250 µg/mL, suggesting a mild proliferative tendency without evidence of cytotoxicity. In contrast, compound **17** exhibited the opposite pattern: lower concentrations induced a modest reduction in viability, whereas higher concentrations resulted in viability values approaching 100%, indicating minimal cytotoxicity at elevated doses. Importantly, the observed increases in viability should not be interpreted as enhanced mitosis but rather as subtle metabolic stimulation within the cells. Overall, these findings support that the tested compounds are noncytotoxic under the evaluated conditions and may differentially modulate cellular metabolic activity depending on concentration.

The comparison between the in silico predictions and the in vitro assays offers additional insight into the structure-activity relationships of these derivatives. Docking analyses predicted moderate to high affinity toward COX-2 for **6**, **10**, and **17**, supported by favorable binding energies and interactions with the catalytic triad residues Arg120, Tyr355, and Ser530. Although the in vitro assays do not directly measure enzymatic inhibition shown in Figure 4, the observed reductions in PGE_2_ production align qualitatively with the computational predictions, particularly for **17**, which showed both the strongest theoretical affinity and the most pronounced PGE_2_ inhibition. However, the correlation is not absolute, reflecting the inherent limitations of docking-based affinity estimation and the biological complexity of macrophage inflammatory responses. However, the combined in silico and in vitro evidence suggests that these derivatives possess a genuine capacity to modulate inflammatory pathways in a manner broadly consistent with the predicted COX-2 interactions.

## 5. Conclusions

This integrative study demonstrates a strong convergence between computational predictions and experimental biological outcomes, supporting phthalimide-based derivatives as promising anti-inflammatory candidates. Molecular docking, supported by DFT optimized geometries, identified plausible binding modes within the COX-2 active site and highlighted key structural determinants governing ligand enzyme recognition, including carbonyl-driven hydrogen bonding and hydrophobic interactions with catalytically relevant residues such as Arg120, Tyr355, and Ser530. Importantly, these in silico results are intended as a structure-based, hypothesis-generating framework to rationalize observed biological trends rather than as definitive evidence of direct enzymatic inhibition.

The biological relevance of these predictions was substantiated through quantitative analysis of PGE_2_ production in LPS-stimulated J774A.1 macrophages using a validated HPLC-based method. Compounds **6**, **10**, and **17** produced a pronounced reduction in PGE_2_ levels, reaching magnitudes comparable to that of the reference drug celecoxib, with compound **17** exhibiting the strongest effect. These results demonstrate a robust anti-inflammatory response consistent with modulation of the COX-2 pathway. Importantly, while reduced PGE_2_ production is functionally aligned with COX-2 involvement, it may also reflect contributions from upstream or downstream regulatory mechanisms within the inflammatory cascade. Accordingly, the observed effects are interpreted as pathway-level modulation rather than definitive proof of direct enzymatic inhibition or absolute COX-2 selectivity.

Equally significant is the favorable safety profile of the evaluated derivatives. All tested compounds maintained cell viability above 90% across the full concentration range, including the highest dose of 250 μg/mL, with no evidence of cytotoxicity or metabolic impairment. This biocompatibility contrasts with the known antiproliferative effects of several precursor fragments in tumor models and highlights the stabilizing role of the phthalimide scaffold in immune cell systems.

Overall, the combined computational and experimental data indicate that strategic hybridization of phthalimide with bioactive aromatic and terpenoid motifs yields compounds capable of effectively attenuating inflammatory signaling associated with PGE_2_ biosynthesis. By integrating structure-based modeling with functional cellular readouts, this work provides a coherent and mechanistically plausible framework for identifying phthalimide derivatives as viable leads for anti-inflammatory drug development. Further enzymatic, mechanistic, and in vivo studies will be essential to fully elucidate their mode of action and to advance these compounds toward therapeutic application.

## Figures and Tables

**Table 1:** Interactions of the ligands with the active site and their binding energies. Highlighted in red: Interaction with residues of the catalytic triad. * Hydrogen bond interaction. ** Two or more hydrogen bond interactions.

Compound	COX-1 ΔG kcal/mol	COX-2 ΔG kcal/mol	COX-1 Residues	COX-2 Residues
**1**	−6.64	−6.51	PHE381, TYR385, LEU384, TRP387, PHE518, VAL349, SER353, ILE523, MET522, GLY526, SER530 *	TYR385, TRP387, LEU384, MET522, VAL523, GLY526, ALA527, VAL349, SER530 *
**2**	−8.2	−8.24	GKY526, SER530, LEU351, VAL116, LEU359, VAL349, TYR355, SER353, LEU352, LEU523, MET522, GLY526, TRP387, TYR385	ARG120 **, VAL523, LEU93, TYR355, LEU359, LEU352, VAL349, SER530 *, LEU531, ALA527
**3**	−9.97	−10.64	LEU359, LEU357, TYR355, LEU93, THR89, VAL116, VAL349, ARG120, SER530, ALA527, GLY526, ILE523, MET522, PHE518, TRP387, LEU384	SER530 *, ALA527, VAL349, LEU359, VAL116, ARG120 *, TRP387, VAL523, TYR355, LEU93, VAL89
**4**	−6.6	−7.71	PRO86, THR89, LEU93, TRP100, LEU112, LEU115, VAL116, VAL119, ARG120, GLU524	GLU524*, PRO86, ARG120, TYR355, LEU93, VAL89, LYS83, VAL116, PHE357, ILE112
**5**	−6.69	−8.59	VAL119, ARG120, VAL116, LEU115, LEU112, LEU357, TRP100, LEU93, THR89, PRO86, ARG83, GLY471, GLU524	GLU524, LEU93, VAL89, PRO84, MET471, LYS83, TYR115, SER119, VAL116, LEU123, ARG120, TYR355
**6**	−9.68	−10.38	VAL116, LEU359, LEU357, TYR355, LEU93, SER353, LEU352, ARG120 *, VAL349, ALA527, ILE523, PHE518, GLY526, TRP387, LEU384, TYR385, SER530, LEU351	ILE112, LEU93, VAL116, TYR355, ARG120 *, SER353, LEU351, ALA527, VAL523, LEU352
**7**	−8.91	−8.23	VAL119, ARG120, VAL116, LEU115, LEU357, TYR355, LEU93, THR89, PRO86, GLU524	LYS89, GLU524 *, PRO86, ARG120, VAL89, LEU93, VAL116, TYR355
**8**	−8.2	−8.21	ARG120, VAL119, LEU123, VAL116, LEU112, LEU357, TYR355, THR89, LEU93, GLU524, GLY471, PHE470	LEU93, PHE357, TYR355, VAL89, PRO86, VAL116, ARG120, GLU524, LYS83, PHE470, MAT471, LEU123
**9**	−6.97	−8.73	VAL116, LEU531, LEU357, VAL349, LEU93, ALA527, ARG120, TYR355 *, SER353 *	VAL116, SER119, ARG120, LYS83, PRO86, VAL89, LEU93, ILE112, TYR355, TYR115
**10**	−7.86	−8.42	LEU534, SER530, PHE205, TYR385, VAL344, TYR348, TRP387, VAL349, LEU352, TYR355, ILE523, ARG120, ALA527	PHE357, ILE112, VAL116, LEU123, LYS83, PHE470, LEU472, GLU524, PRO86, ARG120 *, LEU93, TYR355 *
**11**	−6.62	−9.49	VAL119, ARG120, VAL116, LEU115, LEU112, LEU93, THR89	TYR115, SER119, LYS83, PRO84, LEU123, THR85, VAL89, ARG120 *, PRO86, LEU93, TYR355 *
**12**	−6.02	−8.78	LEU357, LEU112, LEU359, LEU115, VAL116, VAL119, ARG120, THR89, LEU93, LEU92, TYR355	ILE124, HIS122, ARG144, THR60, LYS546, PHE367, LEU366, PHE37, LYS369, GLN372, PHE371, GLN370, SER126, ALA543
**13**	−6.27	−7.62	LEU115, VAL116, VAL119, ARG120, GLU524 *, PRO86, TYR355, THR89, LEU93, LEU357	GLY526, ALA527, LEU531, SER530, ARG120 *, VAL116, SER119, TYR115, ILE112, LEU92, MET113, PHE357, LEU359, VAL349, LEU352
**14**	−8.23	−7.84	PHE205, LEU534, SER530, PHE209, PHE381, TYR385 *, GLY526, TRP387, MET522, PHE518, LEU352, VAL349, TYR348	ASN375, GLY227, ASN537, VAL538, PHE142, ARG376 *, LEU145, GLN374 *
**15**	−7.53	−9.92	SER530, VAL349, ALA527, LEU359, VAL116, LEU351, VAL349, LEU352, SER353, TYR355, LEU93, ARG120	TYR348, TYR385, SER530, ALA527, LEU359, VAL116, ARG120 *, VAL89, LEU93, TYR355, LEU352, LEU384
**16**	−8.06	−8.29	VAL116, LEU359, VAL349, TYR355, SER353, LEU352, LEU351, SER530, ALA527, GLY526, TRP387, MRT522, PHE518, LEU384, TYR385	GLY526, VAL523, HIS90 *, SER353, ALA516, GLN192, PHE518, VAL349, TRP387, TYR385
**17**	−6.05	−6.84	VAL116, LEU123, ARG120, TYR355, LEU93, THR89, PHE470, GLY471, GLU524	VAL116, LEU123, SER119, TYR115, ARG120, GLU524, VAL89, PRO86, PRO84, LYS83, MET471
**Celecoxib**	−6	−9.16	LEU359, LEU357, LEU93, THR89, GLU524, ARG120 *, TYR355, VAL116	VAL116, TYR355, SER353, VAL349, LEU352, TRP387, ALA516, VAL523, HIS90 *, ARG513 *, ALA527, SER530, ARG120
**Diclofenac**	−6.67	−8.73	ARG120, LEU531, VAL349, SER530, LEU352, TRP387, TYR385, TYR348, MET522, PHE518, ILE523, ALA527, GLY526	TYR355, VAL523, LEU352, SER530, LEU384, TRP387, GLY526, MET522, TYR385, PHE381, LEU531, VAL349, ALA527
**Etoricoxib**	−6.64	−8.64	GLU524, THR89, LEU112, TRP100, LEU93, LEU357, LEU359, VAL116, TYR355 *, ARG120 *	LEU531, VAL349, GLY526, ALA527, TRP387, LEU352, VAL523, GLN192, ALA516, PHE518, HIS90, ARG513 *, TYR353, TYR355, VAL116, LEU359, SER530

**Table 2:** Ligand selectivity to COX-2 using Ki = ΔG/RT; deltaG is the docking energy, R is 1.98719 cal/mol-K, and T is 298.15 °K, to selectivity Log_10_ = (K_i_ COX-2/K_i_ COX-1).

Compound	K_i_ COX-1	K_i_ COX-2	Log_10_(K_i_ COX-2/K_i_ COX-1)	Compound	K_i_ COX-1	K_i_ COX-2	Log_10_(K_i_ COX-2/K_i_ COX-1)
**1**	13.62 µM	16.8 µM	0.0911	**11**	14.0 µM	109.86 nM	−2.1052
**2**	977.49 nM	916 nM	0.0282	**12**	38.69 µM	368.07 nM	−2.0216
**3**	49.01 nM	15.83 nM	−0.4908	**13**	25.37 µM	2.59 µM	−0.9910
**4**	14.54 µM	2.24 µM	−0.8123	**14**	824.4 nM	1.79 µM	0.3367
**5**	12.45 µM	505.49 nM	−1.3914	**15**	3.02 µM	53.2 nM	−1.5794
**6**	79.6 nM	24.79 nM	−0.5066	**16**	1.23 µM	836.8 nM	−0.1672
**7**	293.35 nM	926.02 nM	0.4992	**17**	36.62 µM	9.62 nM	−0.6158
**8**	3.04 µM	953.96 nM	−0.5033	**Celecoxib**	39.72 µM	192.37 nM	−2.3148
**9**	7.82 µM	397.25 nM	−1.2941	**Diclofenac**	12.96 µM	400.84 nM	−1.5096
**10**	1.74 µM	675.48 nM	−0.4112	**Etoricoxib**	12.42 µM	465.33 nM	−1.4263

**Table 3:** Cell viability, PGE_2_ production, and inhibition profiles of the evaluated compounds at different concentrations.

Sample	Amount (µg/mL)	% Viability ± SD*	PGE_2_ Production (µg/mL)	% Inhibition ± SD*
**6**	50	97.57 ± 6.52	0.2852	90.66 ± 4.90
**10**	65	106.84 ± 4.26	0.1483	95.22 ± 6.03
**17**	50	85.55 ± 9.90	0.0680	97.79 ± 5.02
Phthalimide	37	91.57 ± 6.26	2.0282	33.56 ± 4.87
Chrysin	13	94.72 ± 3.71	0.8953	70.74 ± 9.91
Geraniol	30	71.63 ± 6.69	0.1733	94.49 ± 3.91
3,4,5-trimethoxybenzoic acid	13	71.77 ± 5.53	0.7986	73.90 ± 6.64
Celecoxib	0.42	NT*	0.0849	97.24 ± 3.07
LPS (+)	5	NT*	3.0544	0
MOS (-)	0	100	ND*	100
DMSO	1	91.28 ± 5.06	NT*	NT*
Curcumin	15	21.22 ± 2.32	NT*	NT*

NT* = not tested, ND* = not detected, SD* = standard deviation, (+) positive control, (-) negative control.

## Data Availability

Data are contained within the article and in the Supplementary Material.

## Supplementary Materials

The following supporting information can be downloaded at https://www.mdpi.com/article/10.3390/pharmaceutics18010129/s1. The Supporting Information file contains graphical representations of the molecular interactions between selected phthalimide derivatives and the active sites of COX-1 and COX-2.
