# Supplementary material for: Phthalimide Derivatives as Anti-Inflammatory Agents: In Silico COX-2 Targeting and In Vitro Inhibition of PGE2 Production
Source: Pharmaceutics. 2026 Jan 20;18(1):129. doi: 10.3390/pharmaceutics18010129 (PMC12845445; doi:10.3390/pharmaceutics18010129)
Supplement: Supplementary file 1 [file pharmaceutics-18-00129-s001.zip › pharmaceutics-4063856-supplementary.pdf]

pharmaceutics

Supporting information

Phthalimide Derivatives as Anti-Inflammatory Agents: In Silico COX-2 Targeting and In Vitro Inhibition of PGE_2 Production

Héctor M Heras Martínez[1], Blanca Sánchez-Ramirez[1], Linda Landeros-Martínez[1], David Rodríguez Guerrero[1], José C. Espinoza-Hicks[1], Gerardo Zaragoza-Galán[1], Alejandro Bugarin[2*] and David Chávez-Flores,[1*]

1 Facultad de Ciencias Químicas, Universidad Autónoma de Chihuahua, Chihuahua 31125, México; p288343@uach.mx (H.M.H.-M.); bsanche@uach.mx (B.S.-R.); lilanderos@uach.mx (L.-L.L.-M.); jhicks@uach.mx (J.C.E.-H.), gzaragoza@uach.mx (G.Z.-G) and a345673@uach.mx (D.R.-G.)

2 Department of Chemistry and Physics, Florida Gulf Coast University, Fort Myers, FL 33965, USA.

* Correspondence: abugarin@fgcu.edu (A.B.); dchavezf@uach.mx (D.C.-F.)

Index:

General Information…… S-1

Figures of interactions COX-1 and COX-2…… S-2

1. General information

The Supporting Information file contains graphical representations of the molecular interactions between selected phthalimide derivatives and the active sites of COX-1 and COX-2. The interaction maps were generated using LigPlot, while molecular structures and visualization were prepared with Open Babel. These figures provide a detailed view of hydrogen bonding and hydrophobic contacts, complementing the docking results discussed in the manuscript. The Supporting Information is provided to give readers a clearer structural perspective on ligand–enzyme interactions and to support the interpretation of the *in-silico* findings.

S2. Figure interactions COX-1 and COX-2

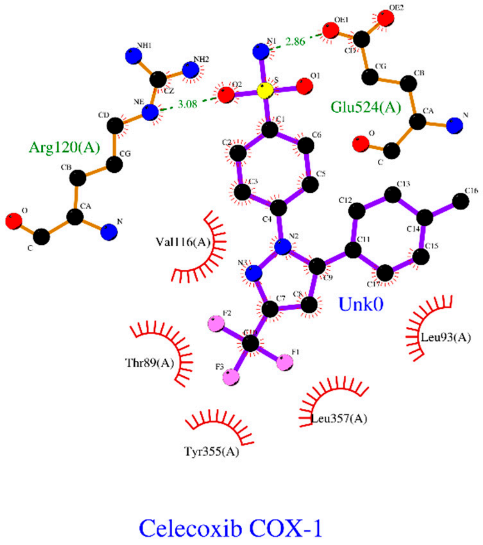

Figure S1. Interaction of Celecoxib with the COX-1 and COX-2 prostaglandin receptor

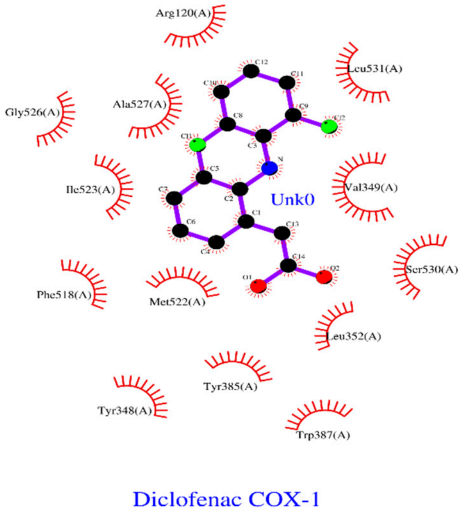

Figure S2. Interaction of Diclofenac with the COX-1 prostaglandin receptor

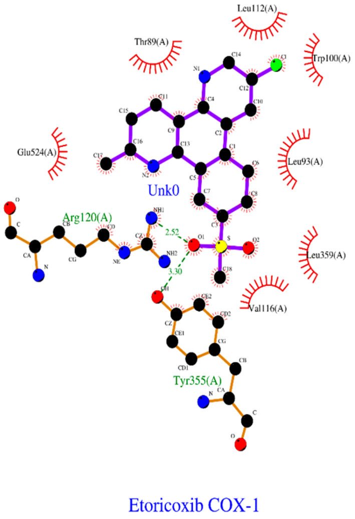

Figure S3. Interaction of Etoricoxib with the COX-1 prostaglandin receptor

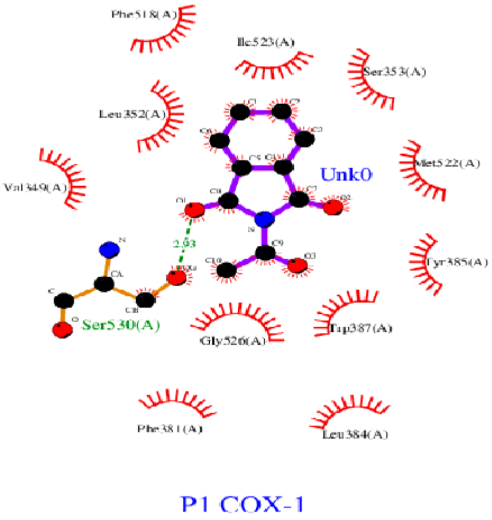

Figure S4. Interaction of **P1** with the COX-1 prostaglandin receptor

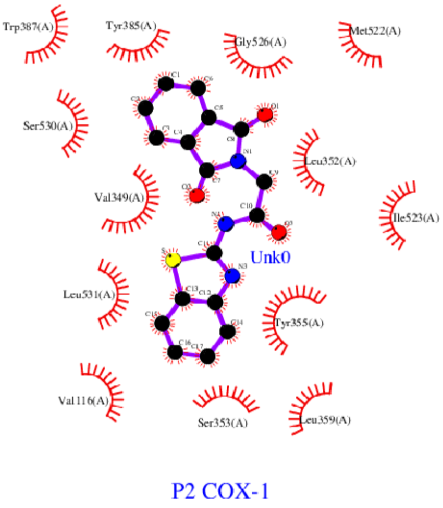

Figure S5. Interaction of **P2** with the COX-1 prostaglandin receptor

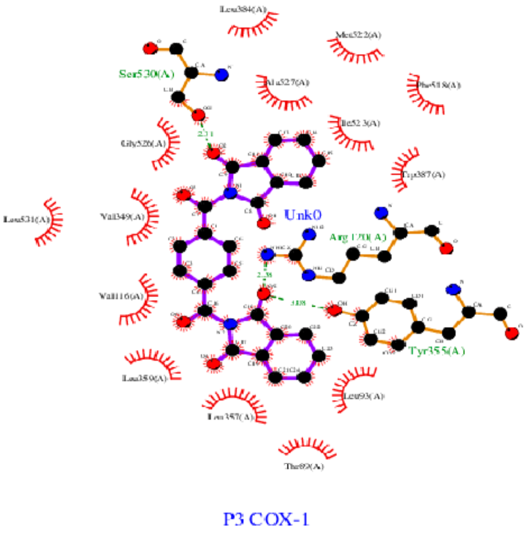

Figure S6. Interaction of **P3** with the COX-1 prostaglandin receptor

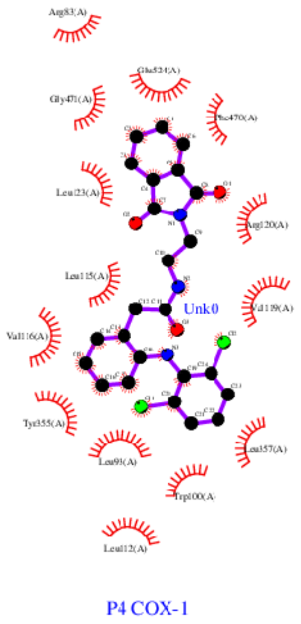

Figure S7. Interaction of **P4** with the COX-1 prostaglandin receptor

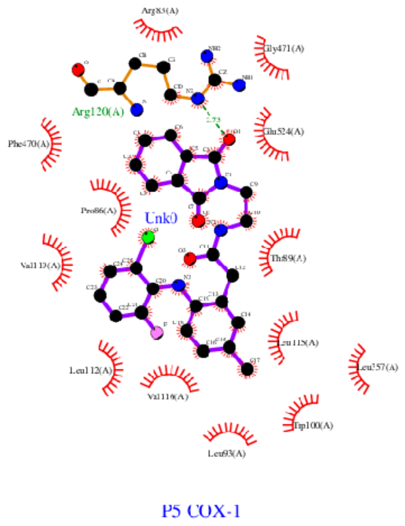

Figure S8. Interaction of **P5** with the COX-1 prostaglandin receptor

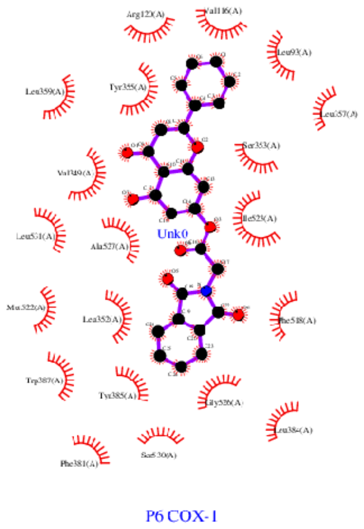

Figure S9. Interaction of **P6** with the COX-1 prostaglandin receptor

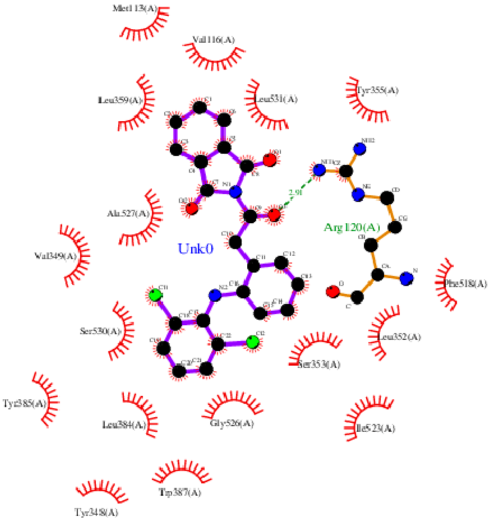

P7 COX-1

Figure S10. Interaction of **P7** with the COX-1 prostaglandin receptor

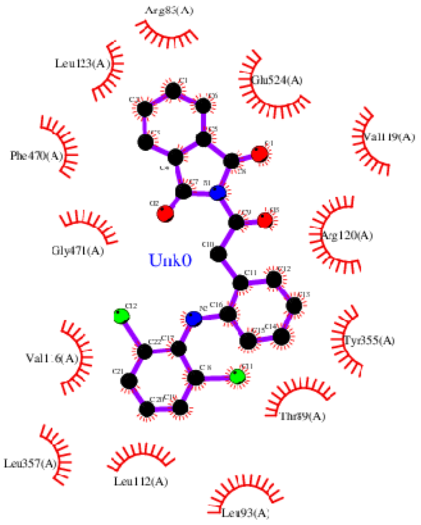

P8 COX-1

Figure S11. Interaction of **P8** with the COX-1 prostaglandin receptor

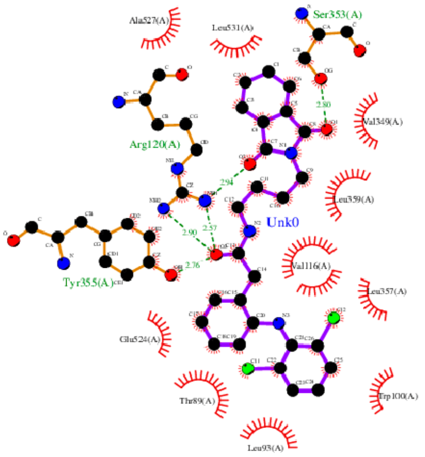

P9 COX-1

Figure S12. Interaction of **P9** with the COX-1 prostaglandin receptor

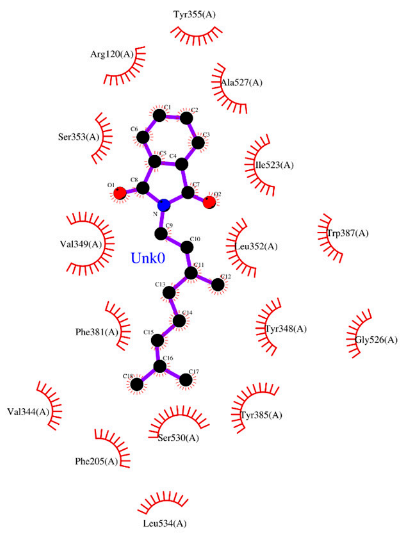

P10 COX-1

Figure S13. Interaction of **P10** with the COX-1 prostaglandin receptor

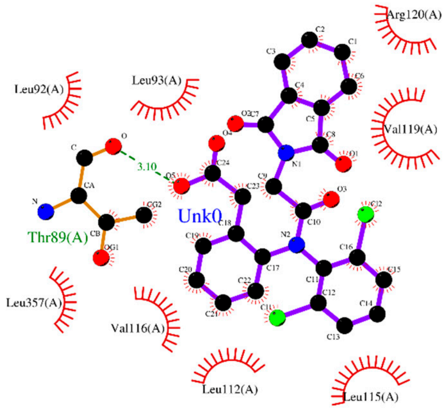

COX1 PDB

Figure S14. Interaction of **P11** with the COX-1 prostaglandin receptor

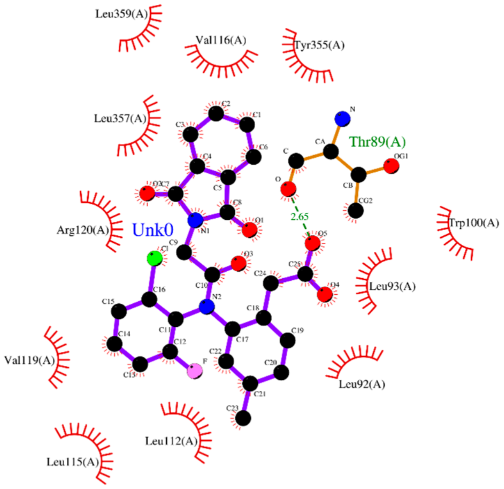

P12 COX1 PDB

Figure S15. Interaction of **P12** with the COX-1 prostaglandin receptor

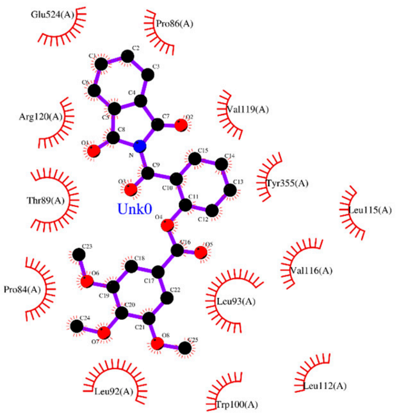

P13 COX-1

Figure S16. Interaction of **P13** with the COX-1 prostaglandin receptor

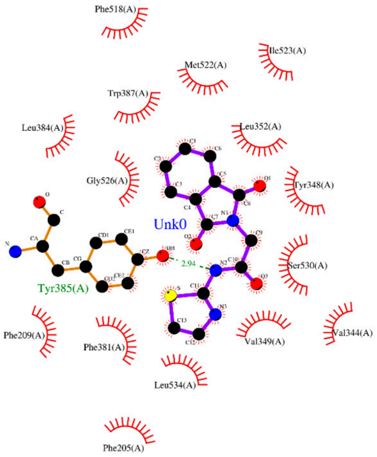

P14 COX-1

Figure S17. Interaction of **P14** with the COX-1 prostaglandin receptor

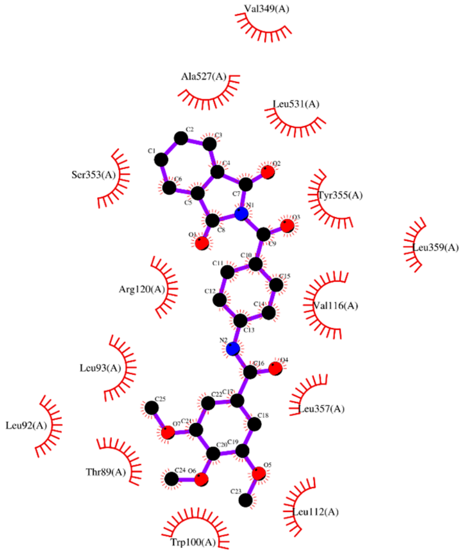

Figure S18. Interaction of **P15** with the COX-1 prostaglandin receptor

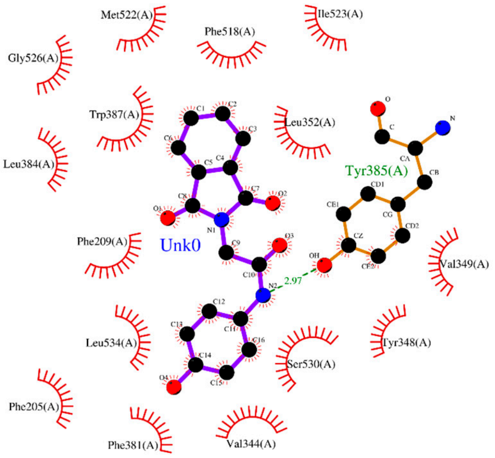

P16 COX-1

Figure S19. Interaction of **P16** with the COX-1 prostaglandin receptor

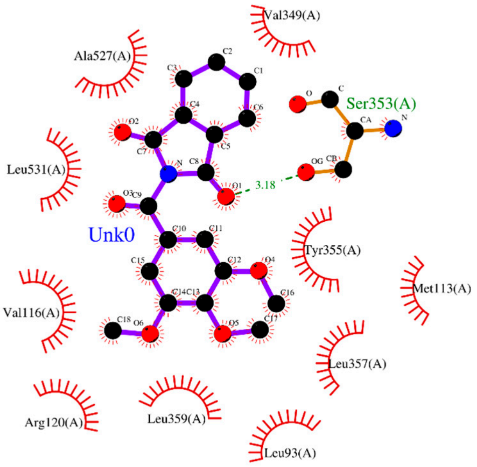

P17 COX-1

Figure S20. Interaction of **P17** with the COX-1 prostaglandin receptor

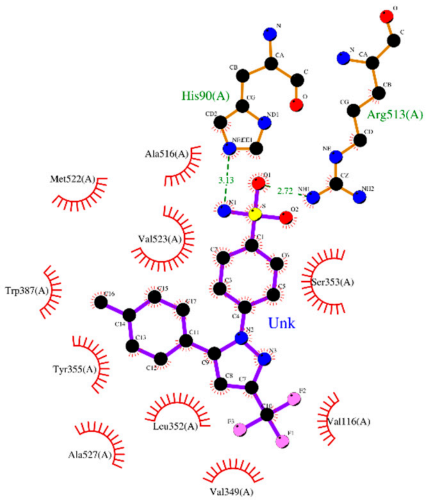

Celecoxib

Figure S21. Interaction of **Celecoxib** with the COX-2 prostaglandin receptor

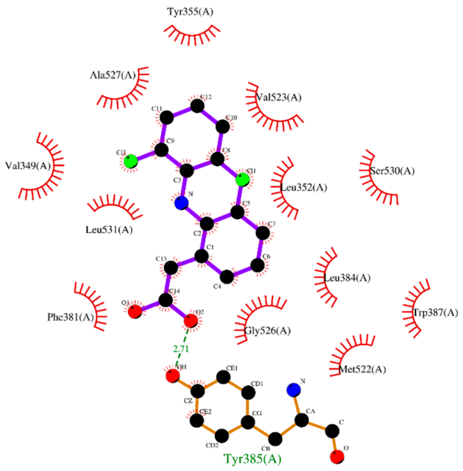

Diclofenaco

Figure S22. Interaction of **Diclofenac** with the COX-2 prostaglandin receptor

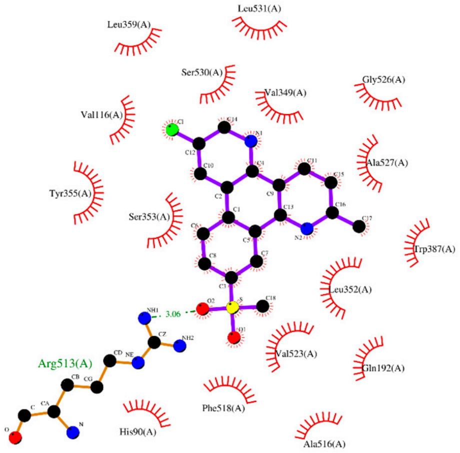

Etoricoxib

Figure S23. Interaction of **Etoricoxib** with the COX-2 prostaglandin receptor

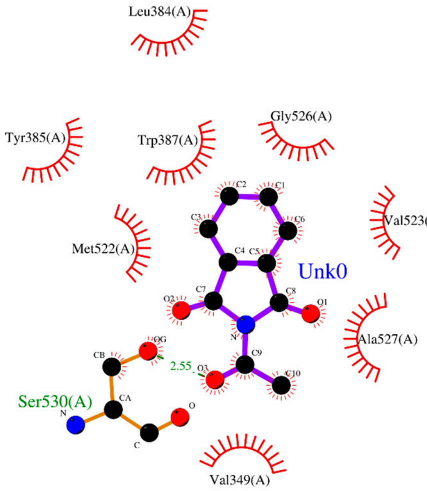

P1 COX-2

Figure S24. Interaction of **P1** with the COX-2 prostaglandin receptor

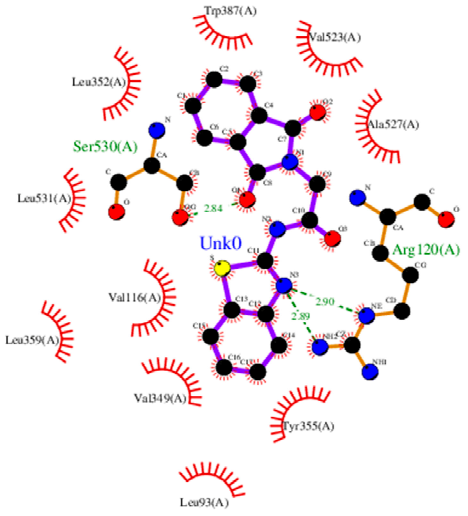

P2 COX-2

Figure S25. Interaction of **P2** with the COX-2 prostaglandin receptor

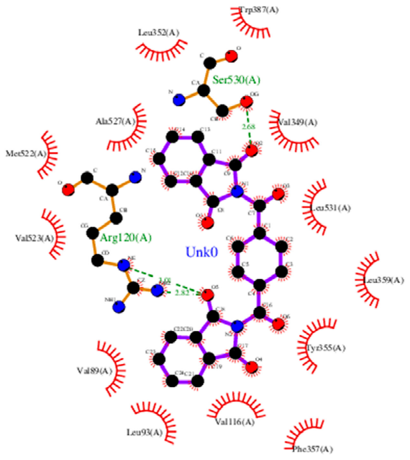

P3 COX-2

Figure S26. Interaction of **P3** with the COX-2 prostaglandin receptor

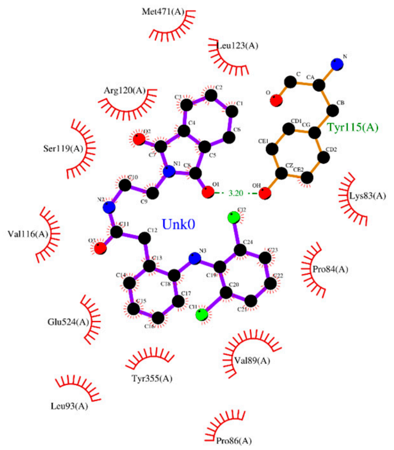

P4 COX-2

Figure S27. Interaction of **P4** with the COX-2 prostaglandin receptor

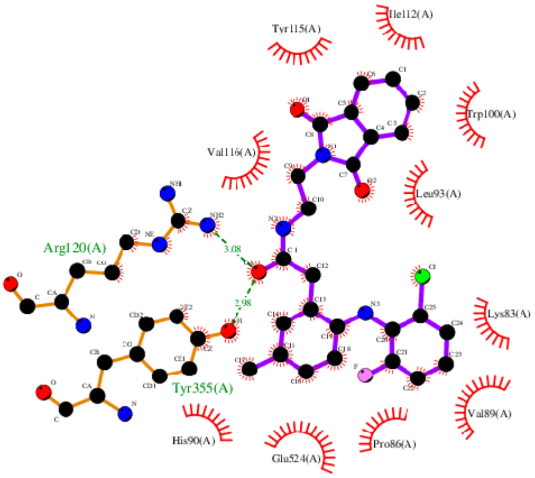

P5 COX-2

Figure S28. Interaction of **P5** with the COX-2 prostaglandin receptor

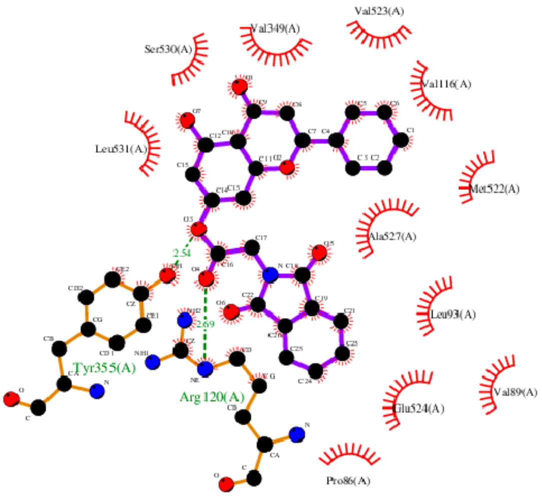

P6 COX-2

Figure S29. Interaction of **P6** with the COX-2 prostaglandin receptor

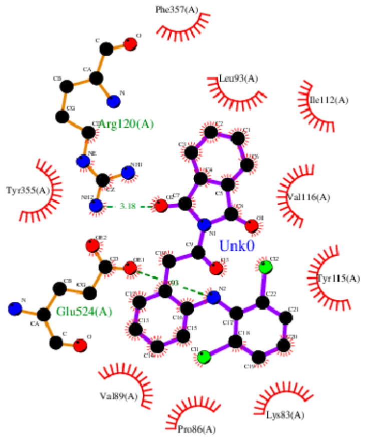

P7 COX-2

Figure S30. Interaction of **P7** with the COX-2 prostaglandin receptor

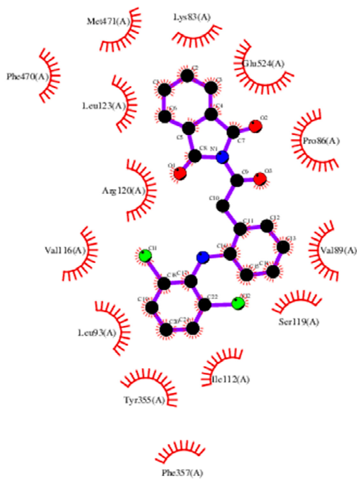

P8 COX-2

Figure S31. Interaction of **P8** with the COX-2 prostaglandin receptor

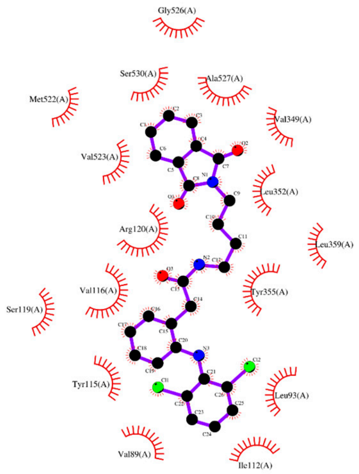

P9 COX-2

Figure S32. Interaction of **P9** with the COX-2 prostaglandin receptor

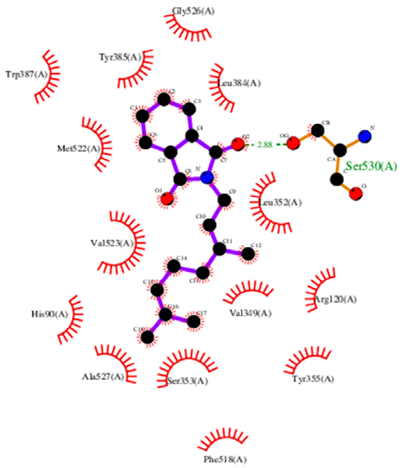

P10 COX-2

Figure S33. Interaction of **P10** with the COX-2 prostaglandin receptor

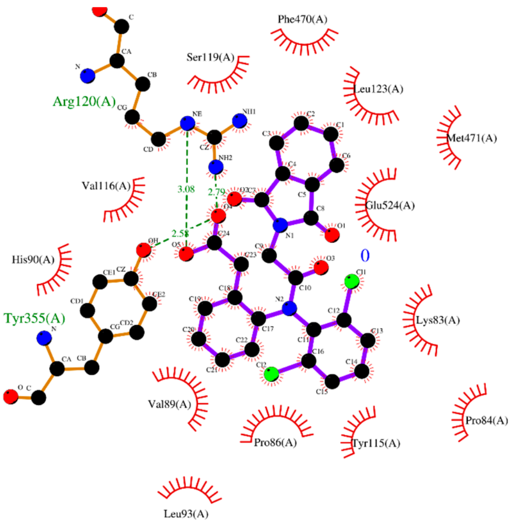

P11 COX2

Figure S34. Interaction of **P11** with the COX-2 prostaglandin receptor

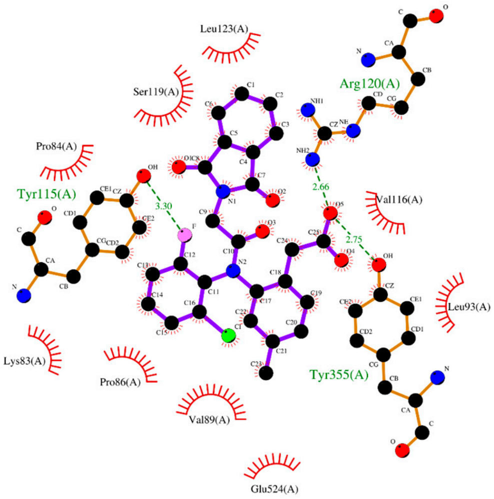

Figure S35. Interaction of **P12** with the COX-2 prostaglandin receptor

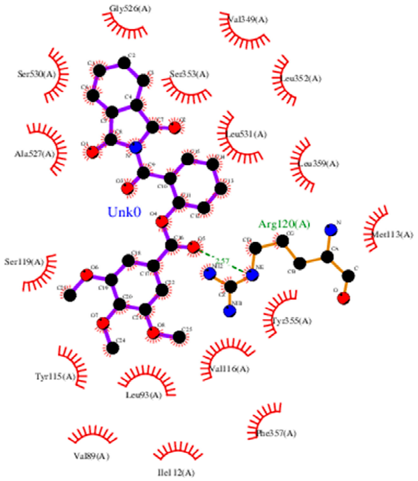

P13 COX-2

Figure S36. Interaction of **P13** with the COX-2 prostaglandin receptor

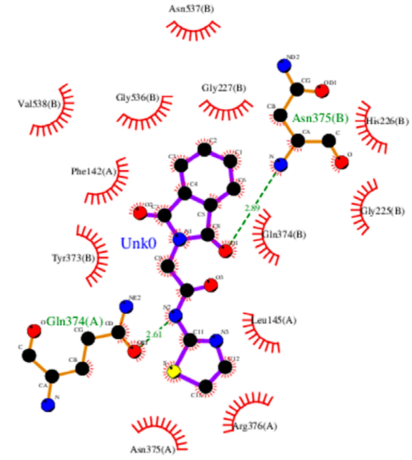

P14 COX-2

Figure S37. Interaction of **P14** with the COX-2 prostaglandin receptor

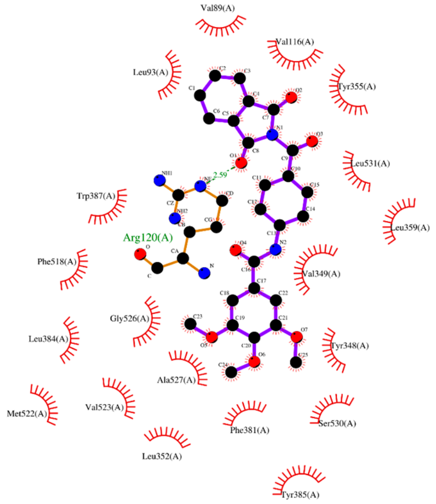

Figure S38. Interaction of **P15** with the COX-2 prostaglandin receptor

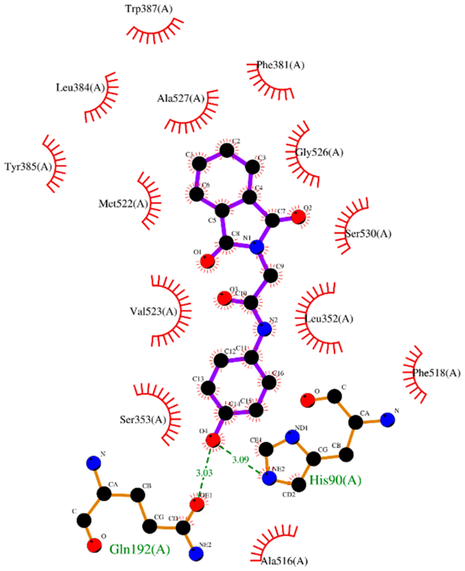

P16 COX-2

Figure S39. Interaction of **P16** with the COX-2 prostaglandin receptor

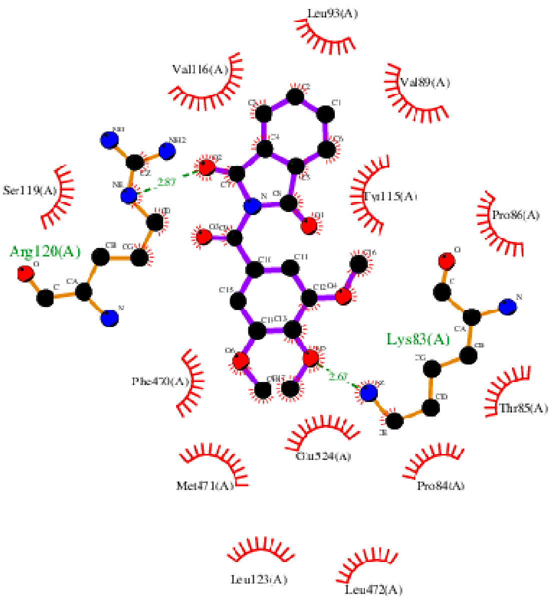

P17 COX-2

Figure S40. Interaction of **P17** with the COX-2 prostaglandin receptor
